# In vitro elution characteristics of gentamicin-impregnated Polymethylmethacrylate: premixed with a second powder vs. liquid Lyophilization

**DOI:** 10.1186/s12891-020-03923-w

**Published:** 2021-01-04

**Authors:** Wongthawat Liawrungrueang, Suwipa Ungphaiboon, Arnurai Jitsurong, Natnicha Ingviya, Boonsin Tangtrakulwanich, Varah Yuenyongviwat

**Affiliations:** 1 Department of Orthopedics, Faculty of Medicine, Prince of Songkla University, Hat Yai, Songkhla 90110 Thailand; 2 Department of Pharmaceutical Technology, Faculty of Pharmaceutical Science, Prince of Songkla University, Hat Yai, Thailand; 3 Forensic Medicine and Toxicology Unit, Department of Pathology, Faculty of Medicine, Prince of Songkla University, Hat Yai, Thailand; 4 Microbiology Unit, Department of Pathology, Faculty of Medicine, Prince of Songkla University, Hat Yai, Thailand

**Keywords:** Antibiotic spacer, Elution characteristics, Lyophilization, Liquid gentamicin

## Abstract

**Background:**

Antibiotic-loaded bone cement, or antibiotic-impregnated polymethylmethacrylate (PMMA), were developed to prevent and treat bone and joint infections. Gentamicin is an antibiotic that is commonly used in combination with PMMA; however, gentamicin powder is hard to obtain in many countries. This study aimed to evaluate the elution characteristics of gentamicin-impregnated PMMA made with lyophilized liquid gentamicin, compared with PMMA; which is made from commercial gentamicin powder.

**Methods:**

The experimental sample was divided into 2 groups: the gentamicin power group (PG-PMMA) and the lyophilized liquid gentamicin group (LG-PMMA). Ten cement spacers were prepared in each group. These were produced by mixing gentamicin powder, or lyophilized liquid gentamicin, with a powder polymer before adding the liquid monomer (2 g of gentamicin and 40 g of PMMA). The volume and surface area of the antibiotic-impregnated cement spacers were 50 cm^3^ and 110 cm^2^, respectively. Each spacer was immersed in phosphate-buffered saline, which was changed daily under sterile conditions. The solutions were collected to measure the level of gentamicin using the enzyme multiplied immunoassay technique (EMIT), at days 1, 2, 3, 4, 5, 6, 7, 14, 21, 28, 35 and 42.

**Results:**

The collections from both groups had high concentrations of gentamicin on day 1 (113.63 ± 23.42 mg/dL in LG-PMMA and 61.7 ±8.37 mg/dL in PG-PMMA), but experienced a continuous decrease over time. The PMMA spacers from both groups could release gentamicin for up to 6 weeks (3.28 ± 1.17 mg/dL in LG-PMMA and 1.21 ± 0.28 mg/dL in PG-PMMA). However, there were significantly higher levels of gentamicin concentrations in the LG-PMMA group compared to the PG-PMMA group at all time points (*P*< 0.05).

**Conclusion:**

Gentamicin-impregnated PMMA made with lyophilized liquid gentamicin had approximately a two times higher rate of antibiotic elution in preliminary in vitro studies, as compared with PMMA made with premixed gentamicin powder.

## Background

Osteomyelitis and periprosthetic joint infections are serious conditions, which lead to significant morbidity, require prolonged treatment and are associated with high rates of recurrence [1, 2]. Antibiotic-loaded bone cement, or antibiotic-impregnated polymethylmethacrylate (PMMA), has been developed to treat chronic osteomyelitis and periprosthetic infections [2]. The benefit of antibiotic-impregnated PMMA is its ability to provide a local concentration of antibiotics to eradicate infection [3], which limits the side effects associated with systemic antibiotics [4]. Gentamicin is one of the antibiotics commonly used to mix with PMMA [1]. If physicians require higher concentrations of gentamicin; when preparing handmade PMMA beads or articulating spacers, gentamicin in powder form is normally used for mixing with PMMA. The reason for this is that most of the available, commercial gentamicin-impregnated PMMA, contains only 0.5–1 g of gentamicin in batches of bone cement (40 g) [5]. However, gentamicin powder is difficult to obtain in some countries where only liquid gentamicin is available [6]. Another drawback of gentamicin powder is its substantially higher cost compared with liquid gentamicin [7]. A previous study reported that gentamicin powder costs approximately 10 times more than liquid gentamicin [2]. Earlier studies have reported the results of gentamicin-impregnated PMMA made with liquid gentamicin [2, 7], and in both of these reports, the dose of gentamicin mixed with a pack of cement was 480 mg. However, preparing handmade gentamicin-impregnated PMMA with liquid gentamicin, in order to obtain a higher concentration of gentamicin, was precluded. As an example; if a physician wanted to add 2 g of gentamicin to 40 g of PMMA, the procedure requires 50 ml of liquid gentamicin [Gentamicin 80 mg/2 ml], so not all of the liquid gentamicin can be mixed with the liquid monomer and cement powder. This is because the high volume of normal saline, which is solvent in liquid gentamicin, will dilute the liquid monomer and prevent the hardening of PMMA. This means that liquid gentamicin cannot be realistically used to produce high-concentrations gentamicin PMMA.

The lyophilization technique, or freeze drying, is a process of dehydration via the lowering of temperature until water freezes; this enables water to change directly from its solid phase to its gas phase. This process is commonplace in procedures of pharmaceutical formulations [8]. However, due to availability limitations, and the high cost of gentamicin powder we believe that lyophilized liquid gentamicin could also be used to make gentamicin-impregnated PMMA. Hence, the aim of this study was to evaluate the elution characteristics of gentamicin-impregnated PMMA made with lyophilized liquid gentamicin, as compared to that of PMMA made with commercial gentamicin powder; using the enzyme multiplied immunoassay technique (EMIT) [9].

## Method

**Fig. 1:**
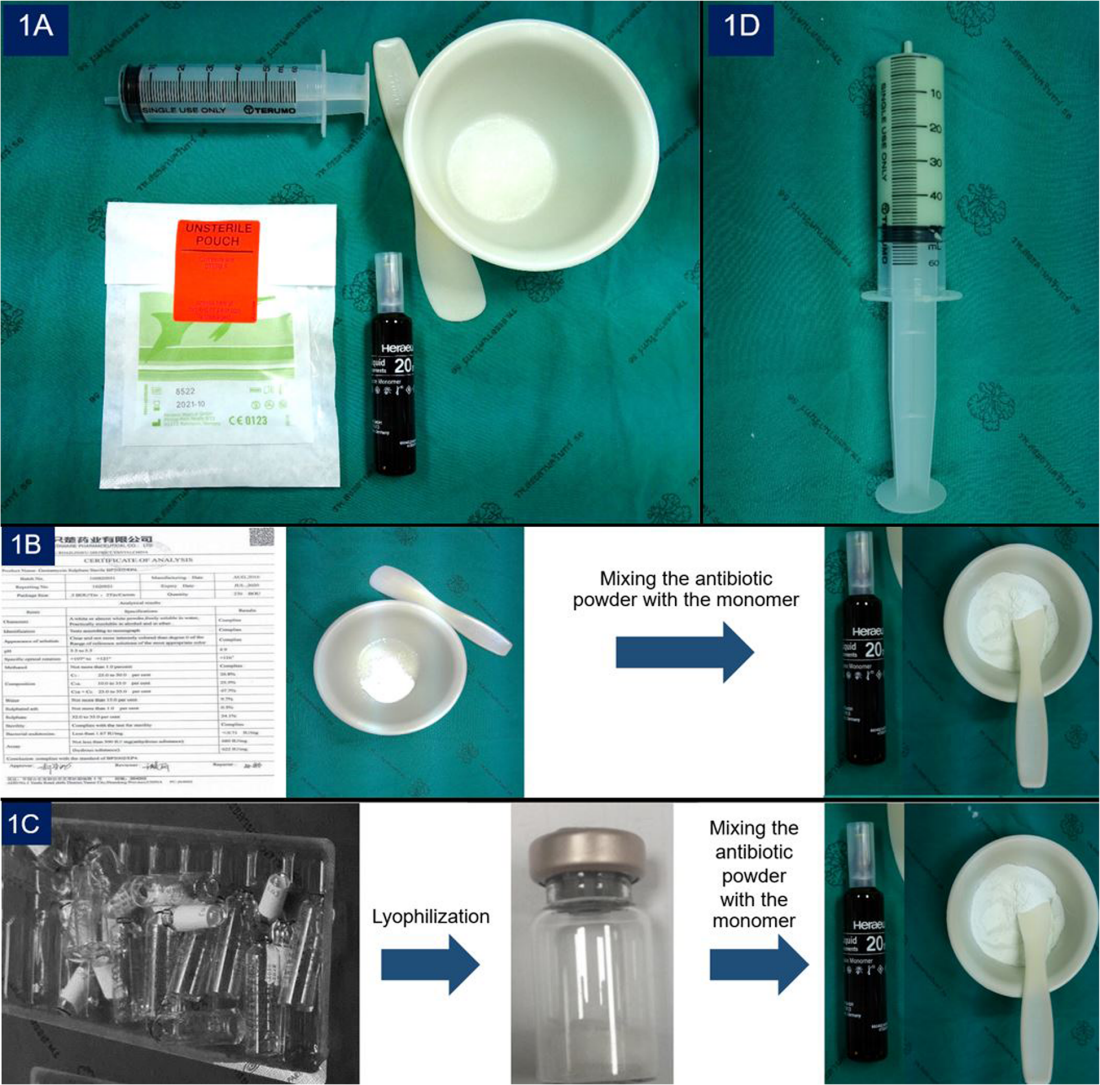
Forty grams of methylmethacrylate polymer (Palacos® R bone cement; Heraeus Kulzer GmbH, Wehrheim, Germany) mixed with 2 g of gentamicin powder (1**a**); 50 ml-syringe filled with the polymer-gentamicin mixture in the dough stage to create the uniformed, cylinder shapes of the cement spacers (1**b**); PG-PMMA spacer group (1**c**) and LG-PMMA spacer group (1**d**)

This was an experimental study that compared the elution properties of gentamicin from cement spacers made with gentamicin powder and lyophilized liquid gentamicin. The research was approved by the Ethics Committee and Institutional Review Board of the Faculty of Medicine, Prince of Songkla University, Thailand. The experimental samples were divided into 2 groups. In the first group, the gentamicin powder group (PG-PMMA), 10 cement spacers were made by mixing 40 g of methylmethacrylate polymer (Palacos® R bone cement; Heraeus Kulzer GmbH, Wehrheim, Germany) with 2 g of gentamicin powder (Gentamicin sulphate; Yantai Justaware Pharmaceutical Ltd., Yantai, China) in a sterile bowl (Fig.1a). The liquid monomer was then added into the bowl. The polymer, gentamicin and the liquid monomer were hand-mixed, and then placed in the pre-defined molds (via 50 ml-syringe) during the dough stage. The volume and surface area of the antibiotic-impregnated cement spacers were 50 cm^3^ and 110 cm^3^, respectively (Fig.1b and c).

The second group, the lyophilized liquid gentamicin group (LG-PMMA), consisting of 10 cement spacers prepared following the same steps as those for the gentamicin powder group; with the exception of using 2 g of lyophilized liquid gentamicin. These were produced by the freeze-drying process (this removes water from a product after it is frozen and placed under a vacuum) for a total of 25 vials of liquid gentamicin [Gentamicin 80 mg/2 ml (1 vial) (Abbott Laboratories, North Chicago, Illinois)]. (Fig.1d).

The cement spacers were removed from the syringe after the cement had set and cooled down. Then each cement spacer was placed in a 150-ml sterile bottle, filled with 100 ml of sterile phosphate-buffered saline (PBS) solution, at a pH of 7.4 (Sigma-Aldrich Corp., St. Louis, Missouri) for preparation of a standard elusion test. All cement spacers were fully immersed in PBS, after which the bottles were covered and then placed in an incubator at 37 °C. The PBS solution in each bottle was changed every day, with 100 mL sterile PBS solution for 6 weeks. On days 1, 2, 3, 4, 5, 6, 7, 14, 21, 28, 35, and 42 the solutions were sent to evaluate their elution of gentamicin. The Enzyme Multiplied Immunoassay Technique (EMIT) was used to evaluate the elution characteristics of gentamicin. A fluorescence polarization immunoassay (FPIA) (Abbot, Wiesbaden, Germany) was used to determine the concentrations of gentamicin released. Both LG-PMMA and PG-PMMA spacers were analyzed with a scanning electron microscope [ver. Quanta 400 (SEM-Quanta)] for surface evaluation [10].

### Statistical analysis

R software version 3.1.0 (R Foundation for Statistical Computing, Vienna, Austria) was used for statistical analysis. The gentamicin concentration between time points in both groups was evaluated using Student t-test. The differences in gentamicin concentration between the groups, along the 6 weeks of study, were evaluated using Generalized Estimating Equations (GEE). A *p*-value less than 0.05 was considered to indicate a statistically significant difference.

## Results

The concentrations of gentamicin from the samples of both groups are shown in Table 1. All of the samples had a high concentration of gentamicin on day 1, which decreased continuously over time. The PMMA spacers from both groups could release gentamicin until the 6th week of study. However, the gentamicin concentrations in the LG-PMMA group were significant higher compared to those of the PG-PMMA group at all time points (*P*< 0.05).

**Table 1:** Mean gentamicin concentration

Samples	Group			*p*-value
	PG-PMMA Mean (SD) (mg/dL)	LG-PMMA Mean (SD) (mg/dL)	Mean difference (SD) (mg/dL)	
Day 1	**61.7 (8.37)**	**113.63 (23.42)**	**51.93 (7.86)**	**< 0.001**
Day 2	**9.26 (1.43)**	**34.04 (5.25)**	**24.77 (1.72)**	**< 0.001**
Day 3	**5.06 (0.49)**	**29.39 (4.49)**	**24.32 (1.43)**	**< 0.001**
Day 4	**4.49 (0.67)**	**22.28 (2.71)**	**17.78 (0.88)**	**< 0.001**
Day 5	**3.63 (0.88)**	**18.81 (3.3)**	**15.18 (1.08)**	**< 0.001**
Day 6	**3.18 (0.67)**	**16.1 (2.55)**	**12.92 (0.83)**	**< 0.001**
Day 7	**3.13 (0.48)**	**14.18 (2.44)**	**11.0.5 (0.76)**	**< 0.001**
Day 14	**2.53 (0.76)**	**7.02 (3.12)**	**4.5 (1.02)**	**< 0.001**
Day 21	**2.05 (0.33)**	**5.9 (1)**	**3.85 (0.33)**	**< 0.001**
Day 28	**1.71 (0.036)**	**5.98 (1.42)**	**4.27 ((0.46)**	**< 0.001**
Day 35	**1.56 (0.29)**	**4.97 (0.79)**	**3.41 (0.27)**	**< 0.001**
Day 42	**1.21 (0.28)**	**3.28 (1.17)**	**2.08 (0.38)**	**< 0.001**

**Fig. 2:**
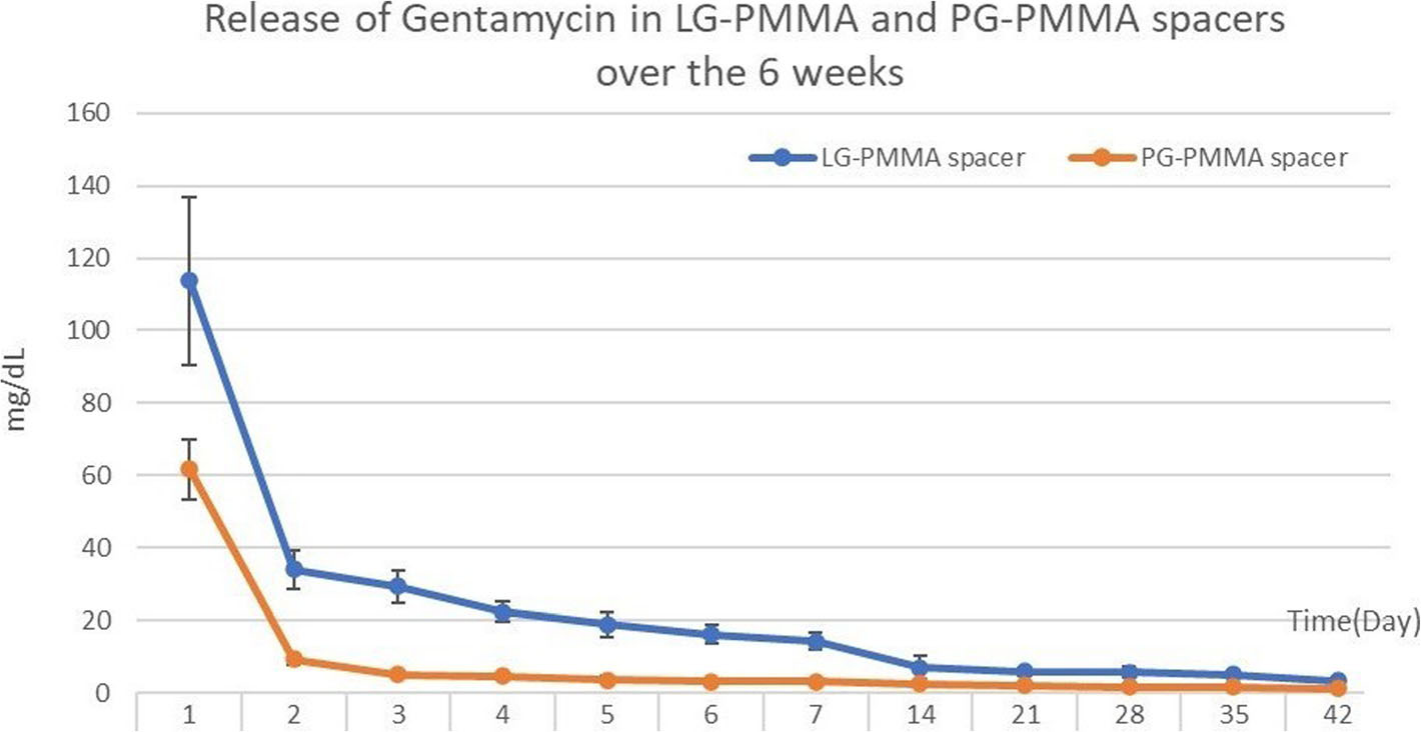
Concentration of eluted gentamicin in LG-PMMA and PG-PMMA groups during the 6-week study period

**Fig. 3:**
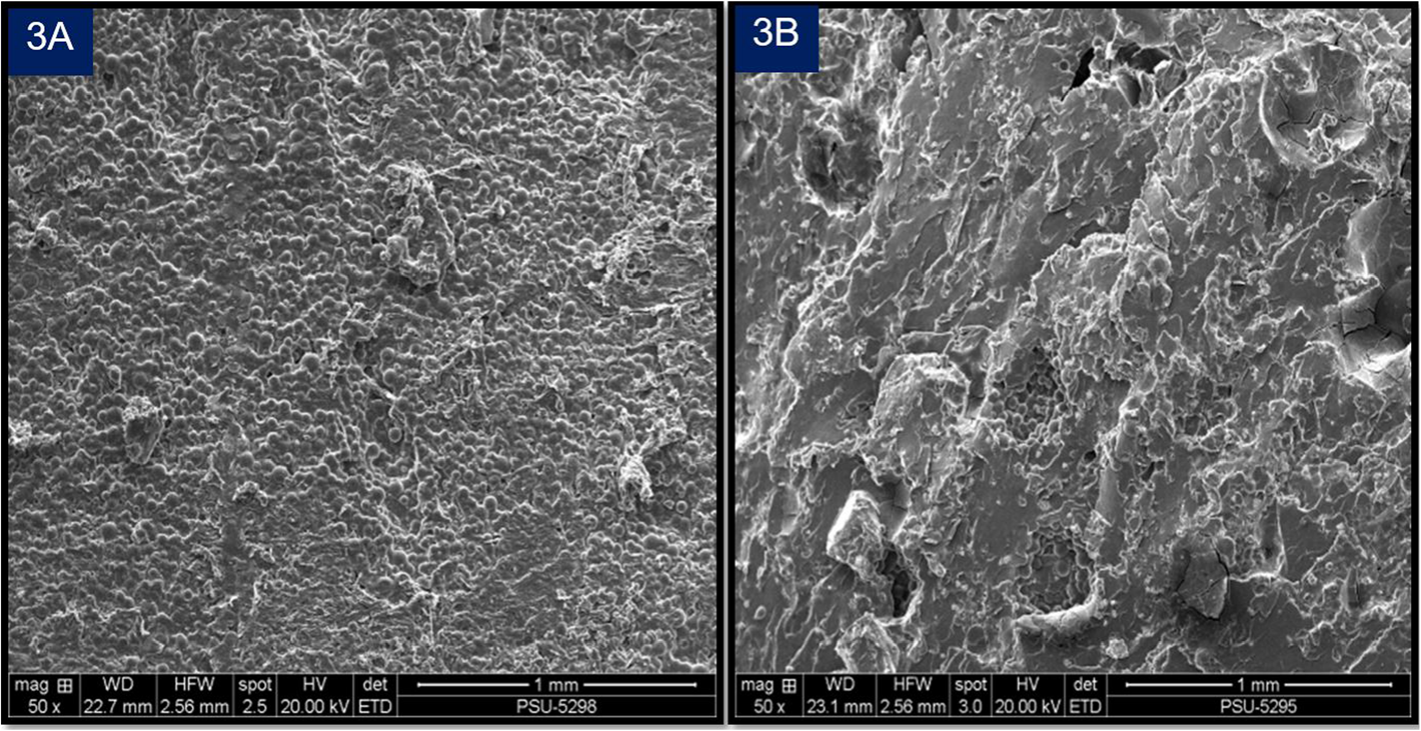
Scanning electron microscope images of the surface of PMMA spacers (3**a**) LG-PMMA spacer; (3**b**) PG-PMMA spacer. The picture of the LG-PMMA spacer showed greater density of small uniformed, rounded shape pores; while the PG-PMMA spacer had a smoother surface area

GEE analysis also showed that the LG-PMMA group showed a significantly higher gentamicin concentration than the PG-PMMA group along the 6-week study period (*p*< 0.05) (Fig.2). Results of the scanning electron microscope (SEM) evaluation indicated that the surface area of the LG-PMMA spacers (Fig. 3a) were more porous than their PG-PMMA counterparts. (Fig. 3b).

**Fig. 4:**
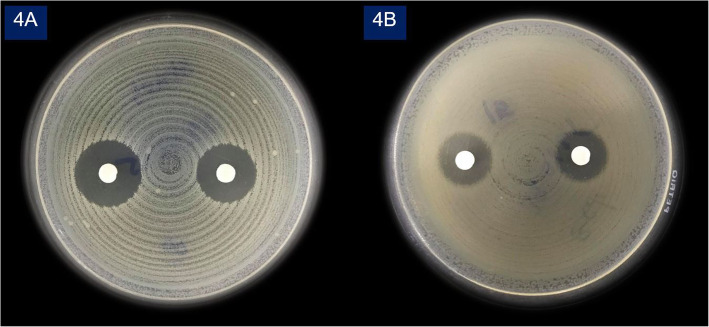
Disk diffusion method for antibiotic susceptibility testing; (4**a**) *Staphylococcus aureus* ATCC 25923, (4**b**) *Escherichia coli* ATCC 25922

## Discussion

The most important benefit of antibiotic-impregnated cement is its ability to provide a high concentration of antibiotics at the local site [11]. Antibiotic-impregnated cement is a treatment option for osteomyelitis and peri-prosthetic infections [2]. Gentamicin is one of the antibiotics commonly used in the preparation of antibiotic PMMA [12–15]. Gentamicin powder is commonly used as a component to produce antibiotic PMMA; however, there is limited availability in certain countries, while the liquid form of gentamicin is more readily available and has a lower cost. Therefore, the authors conducted this research to evaluate the elution characteristics of gentamicin-impregnated PMMA made with lyophilized liquid gentamicin, as compared with PMMA made with commercial gentamicin powder.

This study found that antibiotic-impregnated PMMA, using lyophilized liquid gentamicin, exhibited excellent elution characteristics compared to PMMA produced with gentamicin powder. The reason for this result can be found in previous studies; wherein, a previous report claimed that increasing the porosity of antibiotic-impregnated PMMA increases the antibiotic elution rate [16]. Furthermore, another study reported that adding glycine as a filler in hand-mixed PMMA could increase the elution of gentamicin, by increasing the porosity of the spacer’s surface [17]. We hypothesized that since the liquid gentamicin ampule employed in our study also contained sodium hydroxide, methylparaben, and sodium metabisulfite, after lyophilization, these substances might serve as fillers; and therefore, increase the porosity of LG-PMMA spacers. Our study used a hand mixing technique instead of vacuum-mixing or centrifugation mixing, because these two methods would decrease the porosity of the cement, which in turn would decrease the elution properties of the antibiotics [18].

This study has a number of limitations. First, this study used a hand mixing method that was a potential source of variability; hence, the resultant porosity may vary significantly. It would be more appropriate to use a standardized mixing approach with a device. Therefore, further research in clinical settings is needed to confirm these results before applying them in standard clinical practice. Second, the results from the scanning electron microscope (SEM) were obtained from direct inspection of the surface appearance of the cement spacers; instead of direct measurement of pore size. Finally, this study was only a quantitative study of gentamicin elution without microbiological study. However, the antimicrobial effectiveness of our in-house lyophilized gentamicin was confirmed via disk diffusion method (Agar diffusion test), for antibiotic susceptibility testing Fig. 4. The specific microorganisms in the method were: *Staphylococcus aureus* ATCC 25923 (*S. aureus*) for Gram-positive bacteria (GPB) and *Escherichia coli* ATCC 25922 (E.coli) for Gram-negative bacteria (GNB). The results showed lyophilized gentamicin had an antimicrobial effect. The sterile filter paper discs (6 mm), containing 10 μl of lyophilized gentamicin solution (concentration 39.06 μg /ml), demonstrated a 15 mm inhibition zone for E.coli and an 18 mm inhibition zone for S aureus. This reached the sensitive level (≥ 15 mm) of standard laboratory testing, which used commercial antibiotic discs that contained Gentamicin 10 μg [19]. So, we believe that PMMA spacers made of lyophilized liquid gentamicin should also achieve antimicrobial effectiveness. However, further research for comparing microbiological effects between lyophilized liquid gentamicin with gentamicin powder might be beneficial.

## Conclusion

Gentamicin-impregnated PMMA made with lyophilized liquid gentamicin had approximately a two times higher rate of antibiotic elution in preliminary in vitro studies, comparing with PMMA made from premixed gentamicin powder. This regimen could be utilized in centers with limited availability of premixed gentamicin powder to treat bone and joint infections.

## Data Availability

The datasets generated during this current study are available from the corresponding author upon reasonable request.
